# Could a simple surgical intervention eliminate HIV infection?

**DOI:** 10.1186/1742-4682-1-7

**Published:** 2004-08-31

**Authors:** Slobodan Tepic

**Affiliations:** 1 School of Veterinary Medicine, University of Zurich, Zurich, Switzerland

## Abstract

**Background:**

Human Immunodeficiency Virus (HIV) infection is a dynamic interaction of the pathogen and the host uniquely defined by the preference of the pathogen for a major component of the immune defense of the host. Simple mathematical models of these interactions show that one of the possible outcomes is a chronic infection and much of the modelling work has focused on this state.

**Bifurcation:**

However, the models also predict the existence of a virus-free equilibrium. Which one of the equilibrium states the system selects depends on its parameters. One of these is the net extinction rate of the preferred HIV target, the CD4+ lymphocyte. The theory predicts, somewhat counterintuitively, that *above *a critical extinction rate, the host could eliminate the virus. The question then is how to increase the extinction rate of lymphocytes over a period of several weeks to several months without affecting other parameters of the system.

**Testing the hypothesis:**

Proposed here is the use of drainage, or filtration, of the thoracic duct lymph, a well-established surgical technique developed as an alternative for drug immunosuppression for organ transplantation. The performance of clinically tested thoracic duct lymphocyte depletion schemes matches theoretically predicted requirements for HIV elimination.

## Dynamics of HIV infection and selection of equilibrium states

Reports on the high turnover rates of HIV and its preferred target, CD4+ lymphocytes, during the latent phase of HIV infection [1-3] have established the virus as a prime suspect for direct demolition of the immune system. These clinical findings have also stimulated further efforts at modeling [4,5], and quantitative experimental observation [6]. Major journals have a preference for experimental or clinical data, and the results of mathematical modelling have not reached the broader AIDS research community. For example, the most interesting result of a simple dynamic model published several years ago [7], namely the existence of multiple equilibrium states, one of which is virus-free, has not been discussed in any of the recent publications on HIV response to anti-viral drugs.

For a general medical audience it would be desirable to describe the basic features of the dynamics of HIV infection without recourse to any mathematical formulations. Dynamics implies change over time and the behavior of a dynamic system is defined by stating how the system variables affect each other during a unit of time. In the simplest model there are three system variables: (i) the number of uninfected lymphocytes, (ii) the number of infected lymphocytes and (iii) the number of free virions. The system equations describe how these populations interact. For HIV/CD4+, some of these interactions are understood and generally accepted; others are more speculative, and are subject to further study. However, even with different assumptions about these lesser known aspects, the most interesting result is little affected because it derives from the fact that the rate of infection, i.e. the number of newly infected cells in a unit of time, is proportional to the *product *of the number of uninfected cells and the number of free virions. This makes the resulting equations non-linear, and when the question of equilibrium is addressed, which is done by setting all rates (changes with time) equal to zero, there are two distinct solutions. One of these is free of virus, i.e. the number of virions (and infected cells) is equal to zero, whilst the other equilibrium state has non-zero values for all three populations and thus corresponds to a chronic infection. Which one of the two equilibrium states the system attains depends on the values of the system parameters. The most natural parameter to consider for switching the states is the difference between the rates at which uninfected cells are dying and proliferating. If this parameter, the net extinction rate of healthy lymphocytes, is increased above a critical value, the virus-free equilibrium is selected. This selection (bifurcation) is driven by the conditions of stability; the chronic infection state becomes unstable, i.e. any disturbance takes the system out of it, whilst the virus-free state becomes stable. Once the net extinction rate exceeds the critical value, the system finds its way out of infection. It just so happens that the amount by which the extinction rate needs to be changed, and this based on our current best estimates of other values, is quite modest – several percent of the total lymphocyte population needs to be removed daily.

Depletion of lymphocytes as a therapy for AIDS, based on a population dynamic model, has been advocated by de Boer and Boucher [8]. They proposed that using a suitable immunosuppressant or CD4-killing drug in combination with an anti-viral therapy may eliminate the infection.

This author has arrived at the same result independently using a population dynamics model (three populations, as described above), but also using an expanded model that includes the immune response and, in particular, Tat protein-induced apoptosis [9]. The intervention by lymphocyte depletion will work as predicted by modelling only if other parameters of the system remain substantially unaffected. This is an unlikely outcome with immunosuppressive drugs. Results from limited attempts to use them in HIV-positive patients [10-12] are interesting, but not very encouraging. In fact, the observed rise in CD4+ counts runs *contrary *to the expected effect of depletion.

Activation of latent CD4+ by OKT3 and IL-2 with intention to purge the virus has also been attempted [13], but the outcome was a surprisingly prolonged depletion of CD4+ with little effect on the virus. Our knowledge of the immune system interactions seems inadequate to provide satisfactory explanations for such a response.

As a further illustration of how complex different interventions with biological modifiers can be, treatments with depleting CD4 monoclonal antibody showed a preferential loss of naive T cells, but did not affect IFN-gamma secreting cells [14], providing a clue as to why such depletions did not meet expectations in treating autoimmune diseases.

## Depletion of lymphocytes from the lymphatic circulation

The prediction of the theoretical model calls for the removal of 5 to 10 percent of the total lymphocyte pool per day. The critical value is subject to uncertain estimates of some parameters, and it does differ between the simple, three-parameter and the five-parameter, expanded model. Perhaps the best approach would be to begin depletion, monitor the response by the viral RNA, and then adjust the depletion rate. All of this suggests that some means of physical removal would be best suited. Extracorporeal blood cell separation is a possibility, but the estimate of several hours that the patient would have to spend on the machine daily for several weeks to months, is very discouraging. However, filtration of the thoracic duct lymph, where lymphocytes are present in high concentration, seems almost ideal. The technique of duct drainage for lymphocyte depletion was developed in the sixties and the seventies in order to reduce the risk of organ rejection [15-21]. It has found fairly broad acceptance in renal transplantation [22-25] where the patients would typically be treated for four weeks prior to receiving a transplant. With improved techniques of tissue matching and better immunosuppressive drugs, the thoracic duct drainage lost its appeal in transplant surgery, but it remains an interesting approach to treatments of autoimmune conditions such as rheumatoid arthritis (RA) [26-28]. Improvements in the biocompatibilty of implants could ostensibly even extend the impressive performance of access devices that have remained potent for hundreds of days [29]. The number of lymphocytes removed from RA patients by thoracic duct filtration [29] is in the range of modelling predictions for elimination of the virus (on the order of ten billion per day at the start of the treatment).

An alternative to removal of lymphocytes by duct drainage or filtration is their diversion from the lymphatic system into the gastro-intestinal tract, which has been demonstrated in experimental animals [30-32]. Cells are killed while the precious protein is recycled, avoiding the problem of protein loss by drainage. There is no evidence that HIV could survive gastric passage. The drawback of such a procedure would be in the difficulty of controlling the number of lymphocytes removed. This may not be such a serious limitation, provided the critical value is exceeded. The rate of lymphocyte removal then simply determines the duration of the treatment and the reduction in the number of lymphocytes the patient will experience. This, of course, is an issue that needs careful consideration. Depletion of lymphocytes will cause a transient reduction of their pool (with counts predicted to drop to a few hundred CD4 lymphocytes/microliter), and thus affect the general immune competence. The intervention by depletion should be done as early as possible when the rates of removal necessary are lower and the total pool is less reduced.

## Concluding remarks

Unfortunately, theoretical predictions of system dynamics are not very encouraging for the prospects of HIV vaccines. In principle, the vaccination primes the system for a faster, stronger response, including proliferation of the responding lymphocytes. As the same cells are targets for the virus, the system moves away from the stability condition for the virus-free equilibrium. Apoptosis of uninfected CD4+ lymphocytes in HIV infection is an appropriate response (for the host), albeit insufficient, since the cause of apoptosis is Tat protein produced by only the infected cells themselves. This prevents the system from eliminating the virus because the apoptotic signal *weakens *along with the infection. This suggests a possibility for a pharmacological intervention based on Tat protein that could sustain the apoptotic signal without introducing molecular modifiers with potentially broader effects. If vaccinations were to work, upon entry of the pathogen, they should *provoke *apoptosis of lymphocytes, not their proliferation.

Until such discoveries are made, and to test perhaps their ultimate potential, a simple surgical intervention to allow for removal of lymphocytes merits further investigation. Dangers posed to the patient would be significant, both due to morbidity of the procedure itself, and the expected, but difficult to precisely predict cumulative effects on immunocompetence. An attractive aspect of using physical means of depletion is the possibility to terminate the treatment instantly and completely, as soon as any major deviations from the expected response would arise, indicating a failure of the model and alarming for unexpected risks.

## Competing interests

None declared.

## References

[B1] Wei X, Ghosh SK, Taylor ME, Johnson VA, Emini EA, Deutsch P, Lifson JD, Bonhoeffer S, Nowak MA, Hahn BH, Saag MS, Shaw GM (1995). Viral dynamics in human immunodeficiency virus type 1 infection. Nature.

[B2] Ho DD, Neumann AU, Perelson AS, Chen W, Leonard JM, Markowitz M (1995). Rapid turnover of plasma virions and CD4 lymphocytes in HIV-1 infection. Nature.

[B3] Nowak MA, Bonhoeffer S, Loveday C, Balfe P, Semple M, Kaye S, Tenant-Flowers M, Tedder R (1995). HIV results in the frame. Results confirmed. Nature.

[B4] Coffin JM (1995). HIV population dynamics in vivo; implications for genetic variation, pathogenesis, and therapy. Science.

[B5] Phillips AN (1996). Reduction of the HIV concentration during acute infection: independence from a specific immune response. Science.

[B6] Perelson AS, Neumann AU, Markowitz M, Leonard JM, Ho DD (1996). HIV-1 dynamics in vivo; virion clearance rate, infected cell life-span, and viral generation time. Science.

[B7] Perelson AS, Kirschner D, de Boer R (1993). Dynamics of HIV infection of CD4+ T cells. Math Biosci.

[B8] de Boer RJ, Boucher CAB (1996). Anti-CD4 therapy for AIDS suggested by mathematical models. Proc R Soc Lond B.

[B9] Li CJ, Friedman DJ, Wang C, Metelev V, Pardee AB (1995). Induction of apoptosis in uninfected lymphocytes by HIV-l Tat protein. Science.

[B10] Andrieu JM, Even P, Venet A (1988). Effects of cyclosporin on T-cell subsets in human immunodeficiency virus disease. Clin Immunol Immunopathol.

[B11] Andrieu JM, Lu W, Levy R (1995). Sustained increases in CD4 cells counts in asymptomatic human immunodeficiency virus type 1-seropositive patients treated with predinisolone for 1 year. J Infect Dis.

[B12] Corey L (1995). Editorial: reducing T cell activation as a therapy for human immunodeficiency virus infection. J Infect Dis.

[B13] van Praag RM, Prins JM, Roos MT, Schellekens PT, Ten Berge IJ, Yong SL, Schuitemaker H, Eerenberg AJ, Jurriaans S, de Wolf F, Fox CH, Goudsmit J, Miedema F, Lange JM (2001). OKT3 and IL-2 Treatment for Purging of the Latent HIV-1 Reservoir *in Vivo *Results in Selective Long-Lasting CD4+ T Cell Depletion. J Clin Immunol.

[B14] Rep MH, van Oosten BW, Roos MT, Ader HJ, Polman CH, van Lier RA (1997). Treatment with Depleting CD4 Monoclonal Antibody Results in a Preferential Loss of Circulating Naive T Cells but does not Affect IFN-Gamma Secreting TH1 Cells in Humans. J Clin Invest.

[B15] Joel DD, Sautter JH (1963). Preparation of a chronic thoracic duct venous shunt in calves. Proc Soc Exp.

[B16] Woodruff MFA, Anderson NF (1964). The effect of lymphocyte depletion by thoracic duct fistula and administration of antilympocytic serum on the survival of skin homografts in rats. Ann NY Acad Sci.

[B17] Tilney NL, Murray LE (1968). Chronic thoracic duct fistula: Operative technic and physiologic effects in man. Ann Surg.

[B18] Murray JE, Wilson RE, Tilney NL, Merrill JP, Cooper WC, Birtch AG, Carpenter CB, Hager EB, Dammin GJ, Harrison JH (1968). Five years' experience in renal transplantation with immunosuppresive drugs: survival, function, complications, and the role of lymphocyte depletion by thoracic duct fistula. Ann Surg.

[B19] Fish JC, Mattingly AT, Ritzmann SE, Sarles HE, Remmers AR (1969). Circulating lymphocyte depletion in the calf. Effect on blood and lymph lymphocytes. Arch Surg.

[B20] Sharbaugh RJ, Fitts CT, Majeski JA, Wright FA, Hargest TS (1972). The efficacy of closed-circuit extracorporeal filtration of thoracic duct lymph as a means of lymphocyte depletion. Clin exp Immunol.

[B21] Majeski JA, Fitts CT, Sharbaugh RJ (1977). Architectural alteration of lymphatic tissue produced by extracorporeal thoracic duct filtration. J Surg Oncol.

[B22] Starzl TE, Weil R, Koep LJ, McCalmon RT, Terasaki PI, Iwaki Y, Schroter GP, Franks JJ, Subryan V, Halgrimson CG (1979). Thoracic duct fistula and renal transplantation. Ann Surg.

[B23] Fish JC, Sarles HE, Remmers A, Townsend CM, Bell JD, Flye MW (1981). Renal transplantation after thoracic duct drainage. Ann Surg.

[B24] Starzl TE, Klintmalm GB, Iwatsuki S, Weil R (1981). Late follow-up after thoracic duct drainage in cadaveric renal transplantation. Surg Gynecol Obstet.

[B25] Takeuchi N, Ohshima S, Ono Y, Sahashi M, Matsuura O, Yamada S, Tanaka K, Kuriki O, Kamihira O (1992). Five-year results of thoracic duct drainage in living related donor kidney transplantation. Transplant Proc.

[B26] Paulus HE, Machleder HI, Levine S, Yu DT, MacDonald NS (1977). Lymphocyte involvement in rheumatoid arthritis. Studies during thoracic duct drainage. Arthritis Rheum.

[B27] Vaughan JH, Fox RI, Abresch RJ, Tsoukas CD, Curd JG, Carson DA (1984). Thoracic duct drainage in rheumatoid arthritis. Clin Exp Immunol.

[B28] Sany J (1990). Immunological treatment of rheumatoid arthritis. Clin Exp Rheumatol.

[B29] Sato T, Koga N, Nagano T, Ohteki H, Masuda T, Agishi T (1991). Improved on-line thoracic duct drainage for lymphocytapheresis. Int J Artif Organs.

[B30] Flintoff WM, Tucker HM (1973). Increased homograft survival. Internal thoracic duct-esophageal shunt. Arch Otolaryngol.

[B31] Kawai T, Stoitchcov E, Lorenzini C, Merle M, Benichoux R (1974). Long-term follow-up of dogs, with a patent anastomosis of the thoracic duct to the esophagus. Eur Surg Res.

[B32] Williamson EP, Sells RA (1986). The chyloesophageal fistula. A new approach to thoracic duct drainage. Transplantation.

